# Methods for Reducing Subdivision Error within One Signal Period of Single-Field Scanning Absolute Linear Encoder

**DOI:** 10.3390/s23020865

**Published:** 2023-01-12

**Authors:** Fan Yang, Xinji Lu, Artūras Kilikevičius, Donatas Gurauskis

**Affiliations:** 1 Changchun Institute of Optics, Fine Mechanics and Physics, Chinese Academy of Sciences, Changchun 130033, China; 2 University of Chinese Academy of Sciences, Beijing 100049, China; 3 Department of Mechanical and Material Engineering, Vilnius Gediminas Technical University, LT-03224 Vilnius, Lithuania; 4 Institute of Mechanical Science, Vilnius Gediminas Technical University, LT-03224 Vilnius, Lithuania

**Keywords:** optical encoder, absolute linear encoder, subdivision error within one signal period

## Abstract

Optical encoders are widely used in accurate displacement measurement and motion-control technologies. Based on different measurement methods, optical encoders can be divided into absolute and incremental optical encoders. Absolute linear encoders are commonly used in advanced computer numerical control (CNC) machines. The subdivision error within one signal period (SDE) of the absolute linear encoder is vital to the positioning accuracy and low velocity control of CNC machines. In our paper, we study the working principle of the absolute linear encoder. We proposed two methods for reducing the SDE of the absolute linear encoder, a single-field scanning method based on the shutter-shaped Moiré fringe, as well as a method for suppressing harmonics through a phase shift of index grating. We established a SDE measuring device to determine the absolute linear encoder’s SDE, which we measured using a constant-speed approach. With our proposed methods, the SDE was reduced from ±0.218 μm to ±0.135 μm, which is a decrease of 38.07%. Our fast Fourier transformation (FFT) analysis of the collected Moiré fringe signals demonstrated that the third-, fifth-, and seventh-order harmonics were effectively suppressed.

## 1. Introduction

Optical encoders have the advantages of high accuracy, resolution, and cost performance; fine repeatability; and low environmental sensitivity. Therefore, they are widely used in accurate displacement measurement and motion-control technologies, such as computer numerical control (CNC) machines [1], robotics [2], precision motion-control systems [3], fault diagnosis systems [4], etc. Many methods have been developed to classify optical encoders. Based on the form of displacement measurement, they can be divided into angle and linear encoders. Using different measurement methods, they can be divided into absolute and incremental optical encoders [5]. Absolute linear encoders are widely used in advanced CNC machines. By equipping CNC machines with absolute linear encoders, the absolute position of each axis can be directly obtained without finding the reference position after starting up, and the original processing program can immediately continue following interruption after the power is restarted. This function can greatly improve the processing efficiency of CNC machines. At present, the top manufacturers of absolute linear encoders are: Heidenhain; Fagor; Renishaw; Mitutoyo; Precizika; and Changchun Institute of Optics, Fine Mechanics and Physics.

The position error is the linear encoder’s most important index because it directly determines its performance. The linear encoder’s position error includes the position error over the entire measuring range and the subdivision error within one signal period (SDE) [6]. The position error over the entire measuring range is mainly caused by the imperfections of scale gratings, installation deformations, temperature variation, and vibrations [7]. The scale gratings’ perfectness depends on the lithography machine’s engraving accuracy, and the installation deformations rely upon the mechanical properties of linear encoders and CNC machines. By studying the linear encoder’s thermal expansion coefficient and establishing the appropriate thermal error model, the linear encoder’s thermal error can be compensated [8,9,10,11]. J. Lopez et al. studied the influence of environmental vibrations on the linear encoder’s position error through experiments and finite element analysis, compensating for the linear encoder’s vibration errors [12,13,14]. Cai et al. and Hu et al. proposed an error compensation method based on an empirical mode decomposition (EMD) method for eliminating the influence of environmental factors, such as vibration and temperature variation, on the linear encoder’s position error [15,16].

The SDE is critical not only for positioning accuracy but also for low velocity control; therefore, it is critical for the surface quality of machine parts [6]. The SDE is determined by the linear encoder’s signal period, quality of gratings, and scanning technologies. The smaller the signal period, and the higher the quality of the gratings and scanning technologies, the smaller the SDE. To reduce the optical encoders’ SDE, multiple subdivision algorithms [17,18,19,20] have been proposed, many commercial integrated circuits [21] have been developed, and numerous gratings with special shapes [22,23,24] and devices with special dimensions [25,26] have been applied. Recently, the error compensation models based on particle swarm optimization have been applied to the optical encoders to compensate the SDE [27,28,29]. Although there are many research outcomes, there are few studies on the single-field scanning absolute linear encoder’s SDE.

Our objective in this paper is to examine the single-field scanning absolute linear encoder. We proposed two methods for reducing the absolute linear encoder’s SDE, namely the single-field scanning method based on the shutter-shaped Moiré fringe and the method for suppressing harmonics through a phase shift of the index grating. These two methods do not add additional components and can be easily implemented. In Section 2, we introduce the absolute linear encoder’s working principle and SDE. In Section 3, we perform a theoretical analysis of our two proposed methods for reducing the absolute linear encoder’s SDE. Section 4 provides our experimental results. Finally, we present our conclusions in Section 5.

## 2. Working Principle and SDE of Absolute Linear Encoder

### 2.1. Working Principle of Absolute Linear Encoder

**Figure 1:**
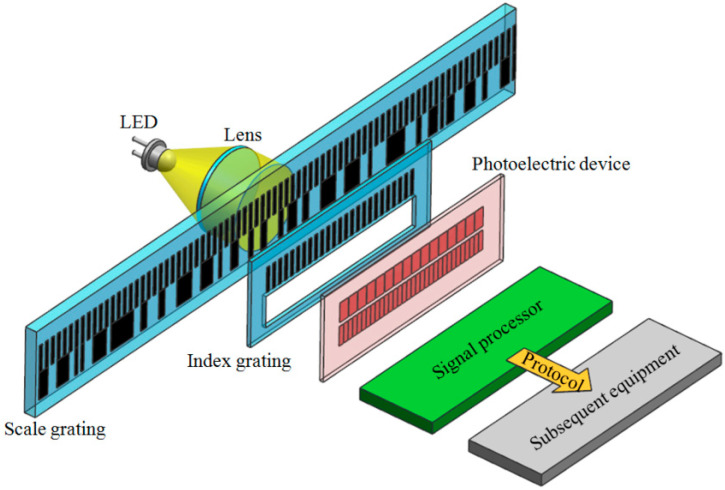
Working principle of absolute linear encoder.

**Figure 2:**
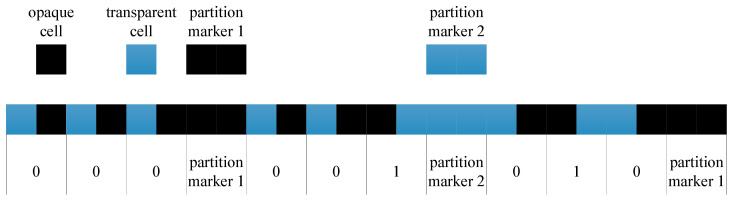
Encoding method of absolute position.

The absolute linear encoder’s working principle is shown in Figure 1. The light emitted by the LED illuminates the scale and index grating after being collimated by the lens. The photoelectric device converts the optical signals into electrical signals. The signal processor calculates the position value and outputs the position value to the subsequent equipment through the communication protocol. The scale grating is composed of two tracks, one of which is an absolute track at the bottom with irregular grid lines. Through this track, the absolute linear encoder’s absolute position information can be obtained in real time. The encoding method of absolute position is based on the binary encoding principle, as shown in Figure 2. The arrangement of transparent and opaque cells represents code “0”, while the arrangement of code “1” is opposite. The advantage of this is that the anti-pollution ability increases. According to the encoding principle, the absolute position code of 000, 001, 010 is illustrated. A partition marker is inserted between each absolute position code to avoid incorrect interpretation of the code. The other track is an incremental track of 20 μm grating on the top with periodic grid lines. Through the Moiré fringe generated by the incremental track and the incremental windows of the index grating, the signals can be highly subdivided, and the resolution of the absolute linear encoder can be improved. Accordingly, the index grating is also composed of two parts. One is a transparent window, corresponding to the absolute track of the scale grating. The other part is incremental windows, engraved with grid lines of 20 μm grating, corresponding to the scale grating’s incremental track. The photoelectric device also consists of two parts: the lower detector receives the absolute signals, while the upper detector receives the incremental signals (Moiré fringe signals). In our research, we adopted single-field scanning technology for the absolute linear encoder. This technology innovatively applies microelectronics integration technology to grating displacement measurements, providing considerable anti-pollution ability and high output-signal quality. All-optical encoding schemes in which no physical gratings are needed are possible. Multiple photon energies are modulated by use of surface plasmons [30]. The encoding in such cases is through amplitude modulation of the pump beam. The encoded signal is the output of the detector.

### 2.2. SDE of Absolute Linear Encoder

The linear encoder’s SDE is determined by the signal period, quality of the gratings, and scanning technologies. As for the absolute linear encoder, the SDE is mostly determined by the incremental signals. The smaller the period of the incremental signals, the smaller the SDE. In our paper, we only analyzed the absolute linear encoder’s signals from the incremental part.

The absolute linear encoder’s incremental signals can be expressed as:

(1) $$\left\{\begin{array}{l} S_{A}=A\sin\left(\frac{2\pi }{d}x\right)\\ S_{B}=A\cos\left(\frac{2\pi }{d}x\right) \end{array}\right.$$

where x is the position value, d is the grating period of the incremental track, and A is the amplitude of the signal. The amplitudes of the two signals are equal, the phase difference is π/2, and there is no DC offset and higher-order harmonics.

According to Equation (1), the position value of x can be calculated as:

(2) $$x_{ideal}=\frac{d}{2\pi }\arctan\left(\frac{S_{A}}{S_{B}}\right)$$

However, due to the errors in optical, mechanical, and electronic systems, the signals generated by the absolute linear encoder are not ideal. The signals have DC offset, amplitude, and phase-shift errors, as well as harmonic distortion in practice, which can be expressed as:

(3) $$\left\{\begin{array}{l} S_{A}{}^{\prime }=a_{0}+\sum_{n=1}^{\infty }A_{n}\sin\left(\frac{2n\pi }{d}x+\varphi_{n}\right)\\ S_{B}{}^{\prime }=b_{0}+\sum_{n=1}^{\infty }B_{n}\cos\left(\frac{2n\pi }{d}x+\psi_{n}\right) \end{array}\right.$$

where $a_{0}$ and $b_{0}$ denote DC offset, n denotes the harmonic order, $A_{n}$ and $B_{n}$ denote the amplitudes of the nth harmonic, and $\varphi_{n}$ and $\psi_{n}$ denote the phase shift of the nth harmonic.

According to Equation (3), x can be calculated as:

(4) $$x_{real}=\frac{d}{2\pi }\arctan\left(\frac{S_{A}{}^{\prime }}{S_{B}{}^{\prime }}\right)$$

In this case, the absolute linear encoder’s SDE is given by:

(5) $$\Delta x=x_{real}-x_{ideal}$$

The DC offset, amplitude, and phase-shift errors, as well as the harmonic distortion of the signals, affect the absolute linear encoder’s SDE.

## 3. Methods for Reducing SDE of Absolute Linear Encoder

Here, we propose two methods for reducing the absolute linear encoder’s SDE: a single-field scanning method based on the shutter-shaped Moiré fringe, and a method for suppressing harmonics through a phase shift of the index grating.

### 3.1. Single-Field Scanning Method Based on Shutter-Shaped Moiré Fringe

**Figure 3:**
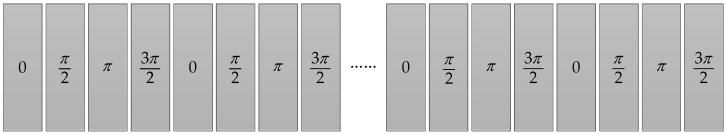
Phase arrangement of longitudinal Moiré fringe signals received by photoelectric device.

The single-field scanning linear encoder usually adopts a longitudinal Moiré fringe, which means the index grating’s incremental windows are fixed. Ideally, the phases of the Moiré fringe signals received by the photoelectric device are 0, π/2, π and 3π/2, respectively, and this arrangement is shown in Figure 3. When d=20 μm and A=1 V, by performing subtractions between Moiré fringe signals with a phase difference of π, we obtain the following signals:

(6) $$S_{A}=\sin\left(\frac{2\pi }{20}x\right)-\sin\left(\frac{2\pi }{20}x+\pi \right)=2\sin\left(\frac{2\pi }{20}x\right)$$

(7) $$S_{B}=\sin\left(\frac{2\pi }{20}x+\frac{\pi }{2}\right)-\sin\left(\frac{2\pi }{20}x+\frac{3\pi }{2}\right)=2\sin\left(\frac{2\pi }{20}x+\frac{\pi }{2}\right)$$

However, due to the divergence of light source, temperature variation, and the engraving and copying of the index grating, linear errors are inevitably introduced, resulting in the phases of Moiré fringe signals with values of 0, π/2+σ, π+2σ and 3π/2+3σ, respectively. Then, the signals can be described by the following expression:

(8) $$S_{A}{}^{\prime }=\sin\left(\frac{2\pi }{20}x\right)-\sin\left(\frac{2\pi }{20}x+\pi +2\sigma \right)=2\cos\left(\sigma \right)\sin\left(\frac{2\pi }{20}x+\sigma \right)$$

(9) $$S_{B}{}^{\prime }=\sin\left(\frac{2\pi }{20}x+\frac{\pi }{2}+\sigma \right)-\sin\left(\frac{2\pi }{20}x+\frac{3\pi }{2}+3\sigma \right)=2\cos\left(\sigma \right)\sin\left(\frac{2\pi }{20}x+\frac{\pi }{2}+2\sigma \right)$$

By comparing Equation (6) with (8), we observed that the signal phase shifts from 0 to σ, and the amplitude changes from 2 V to $2\cos\left(\sigma \right)$ V. By comparing Equation (7) with (9), we observed that the signal phase shifts from π/2 to π/2+2σ, and the amplitude changes from 2 V to $2\cos\left(\sigma \right)$ V. The phases and amplitudes of the two signals changed; therefore, the phase difference was no longer π/2 but π/2+σ. This led to phase-shift error. Therefore, the linear errors will reduce the orthogonality of the two signals and increase the absolute linear encoder’s SDE.

**Figure 4:**
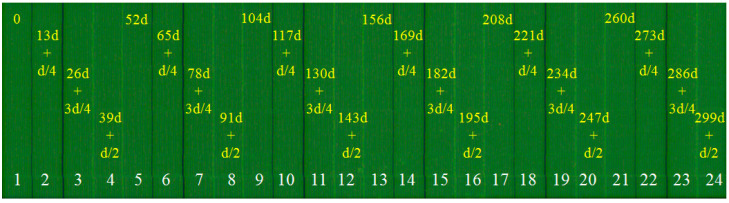
Arrangement of incremental windows of index grating.

The single-field scanning absolute linear encoder that we studied adopted the shutter-shaped Moiré fringe. The influence of linear errors on the quality of Moiré fringe signals is reduced by modifying the arrangement of the incremental windows of the index grating. Figure 4 shows the arrangement of the index grating’s incremental windows. The 24 grating patterns correspond to the 24 pixels of the single-filed scanning photoelectric device. The number of each grating pattern is shown in white at the bottom of Figure 4, and the size of each grating pattern is 0.26 mm (width) × 1.6875 mm (height). The grating period of d is 20 μm. The yellow numbers in each grating pattern indicate the starting position, and the first, second, third, and fourth grating patterns begin with 0, 13d+d/4, 26d+3d/4, and 39d+d/2, respectively, and so forth. The four grating patterns form a group. The distance between the starting positions of two adjacent groups is 52d. The four grating patterns in other groups are arranged in a similar way.

**Figure 5:**
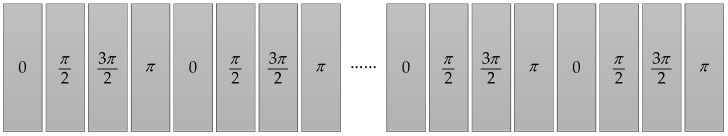
Phase arrangement of shutter-shaped Moiré fringe signals received by photoelectric device.

After modifying the arrangement of the index grating’s incremental windows according to Figure 4, ideally, the phases of the Moiré fringe signals received by the photoelectric device are 0, π/2, 3π/2, and π, respectively. This arrangement is shown in Figure 5.

Linear errors led to the phases of Moiré fringe signals being 0, π/2+σ, 3π/2+2σ, and π+3σ, respectively. Then, the signals are expressed as:

(10) $$S_{A}{}^{\prime }=\sin\left(\frac{2\pi }{20}x\right)-\sin\left(\frac{2\pi }{20}x+\pi +3\sigma \right)=2\cos\left(1.5\sigma \right)\sin\left(\frac{2\pi }{20}x+1.5\sigma \right)$$

(11) $$S_{B}{}^{\prime }=\sin\left(\frac{2\pi }{20}x+\frac{\pi }{2}+\sigma \right)-\sin\left(\frac{2\pi }{20}x+\frac{3\pi }{2}+2\sigma \right)=2\cos\left(0.5\sigma \right)\sin\left(\frac{2\pi }{20}x+\frac{\pi }{2}+1.5\sigma \right)$$

By comparing Equation (6) with (10), we observed that the signal phase shifts from 0 to 1.5σ, and the amplitude changes from 2 V to $2\cos\left(1.5\sigma \right)$ V. By comparing Equation (7) with (11), we discerned that the signal phase shifts from π/2 to π/2+1.5σ, and the amplitude changes from 2 V to $2\cos\left(0.5\sigma \right)$ V. The phases and amplitudes of the two signals changed, and the amplitudes were no longer the same. This led to amplitude error. Therefore, the linear errors will reduce the property of equal amplitude in the two signals and increase the absolute linear encoder’s SDE.

**Figure 6:**
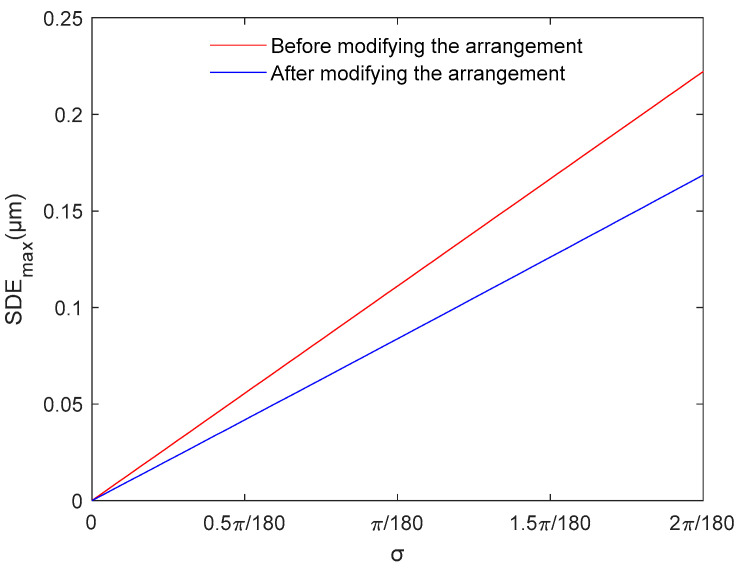
Maximum subdivision error within one signal period (SDE) caused by linear errors.

The maximum SDE caused by linear errors under the two arrangements is compared in Figure 6. After modifying the arrangement of the index grating’s incremental windows, the maximum SDE considerably decreased. When σ=π/180, the maximum SDE decreases from 0.111 μm to 0.084 μm, which is a 24.32% reduction. When σ=2π/180, the maximum SDE decreases from 0.222 μm to 0.169 μm, which is a 23.87% reduction. Because σ can be easily controlled within 2π/180, the maximum SDE caused by linear errors considerably decreases after modifying the arrangement of the index grating’s incremental windows.

The above analysis indicates that modifying the arrangement of the index grating’s incremental windows in the single-field scanning method based on the shutter-shaped Moiré fringe can reduce the influence of linear errors on the Moiré fringe signals phases. Although the amplitude error increases, the SDE decreases.

### 3.2. Method for Suppressing Harmonics through Phase Shift of Index Grating

In our research, we studied the method for suppressing harmonics through a phase shift of the index grating. By designing light and dark fringes with different phases on the index grating and utilizing the signal accumulation effect of the photoelectric device, higher-order harmonics in Moiré fringe signals are suppressed, which reduces the absolute linear encoder’s SDE. Compared with traditional methods, this method does not add additional components and has various advantages, such as a low cost and high real-time performance.

According to Section 2.2, the signals output by the absolute linear encoder are not ideal. The actual single signal can be expressed as:

(12) $$S(x)=a_{0}+\sum_{n=1}^{\infty }A_{n}\sin\left(\frac{2n\pi }{d}x+\varphi_{n}\right)$$

The lower the harmonic order, the greater the influence on the sine of the signals, and the higher the harmonic order, the smaller the impact. Because the proportion of harmonics above the seventh order is negligible, Equation (12) can be simplified as:

(13) $$S(x)=a_{0}+\sum_{n=1}^{7}A_{n}\sin\left(\frac{2n\pi }{d}x+\varphi_{n}\right)$$

Move the x in Equation (13) to the left and right by $\Delta_{1}=d/12$, respectively, and then add the two acquired equations together; then, we have:

(14) $$\begin{array}{c} S_{1}(x)=2a_{0}+\sqrt{3}A_{1}\sin\left(\frac{2\pi }{d}x+\varphi_{1}\right)+A_{2}\sin\left(\frac{2\pi }{d}2x+\varphi_{2}\right)-A_{4}\sin\left(\frac{2\pi }{d}4x+\varphi_{4}\right)-\\ \sqrt{3}A_{5}\sin\left(\frac{2\pi }{d}5x+\varphi_{5}\right)-2A_{6}\sin\left(\frac{2\pi }{d}6x+\varphi_{6}\right)-\sqrt{3}A_{7}\sin\left(\frac{2\pi }{d}7x+\varphi_{7}\right) \end{array}$$

According to Equation (14), the third-order harmonic is suppressed.

Similarly, move the x in Equation (14) to the left and right by $\Delta_{2}=d/20$, respectively, and then add them together to suppress the fifth-order harmonic:

(15) $$\begin{array}{c} S_{2}(x)=4a_{0}+2\sqrt{3}\cos\left(\frac{\pi }{10}\right)A_{1}\sin\left(\frac{2\pi }{d}x+\varphi_{1}\right)+2\cos\left(\frac{\pi }{5}\right)A_{2}\sin\left(\frac{2\pi }{d}2x+\varphi_{2}\right)-2\cos\left(\frac{2\pi }{5}\right)A_{4}\sin\left(\frac{2\pi }{d}4x+\varphi_{4}\right)-\\ 4\cos\left(\frac{3\pi }{5}\right)A_{6}\sin\left(\frac{2\pi }{d}4x+\varphi_{6}\right)-2\sqrt{3}\cos\left(\frac{7\pi }{10}\right)A_{7}\sin\left(\frac{2\pi }{d}7x+\varphi_{7}\right) \end{array}$$

Similarly, move the x in Equation (15) to the left and right by $\Delta_{3}=d/28$ respectively, and then add them together to suppress the seventh-order harmonic:

(16) $$\begin{array}{c} S_{3}(x)=8a_{0}+4\sqrt{3}\cos\left(\frac{\pi }{10}\right)\cos\left(\frac{\pi }{14}\right)A_{1}\sin\left(\frac{2\pi }{d}x+\varphi_{1}\right)+4\cos\left(\frac{\pi }{5}\right)\cos\left(\frac{\pi }{7}\right)A_{2}\sin\left(\frac{2\pi }{d}2x+\varphi_{2}\right)-\\ 4\cos\left(\frac{2\pi }{5}\right)\cos\left(\frac{2\pi }{7}\right)A_{4}\sin\left(\frac{2\pi }{d}4x+\varphi_{4}\right)-8\cos\left(\frac{3\pi }{5}\right)\cos\left(\frac{3\pi }{7}\right)A_{6}\sin\left(\frac{2\pi }{d}6x+\varphi_{6}\right)\; \end{array}$$

Equation (16) shows that odd harmonics (the third-, fifth-, and seventh-order) are suppressed. Except for the fundamental wave, there are only DC offset and even harmonics (the second-, fourth- and sixth-order). In the grating displacement measurement system, four-phase Moiré fringe signals are usually generated: 0, π/2, π and 3π/2. Even harmonics can be suppressed via subtractions between Moiré fringe signals with phase difference of π. We moved the x in Equation (16) to the right by a distance of d/2, and subtract the acquired equation from (16). Then, the signal can be expressed as:

(17) $$S(x)=8\sqrt{3}\cos\left(\frac{\pi }{10}\right)\cos\left(\frac{\pi }{14}\right)A_{1}\sin\left(\frac{2\pi }{d}x+\varphi_{1}\right)$$

From Equation (17), we can see that both DC offset and higher-order harmonics are suppressed. It is an ideal sinusoidal signal.

**Figure 7:**
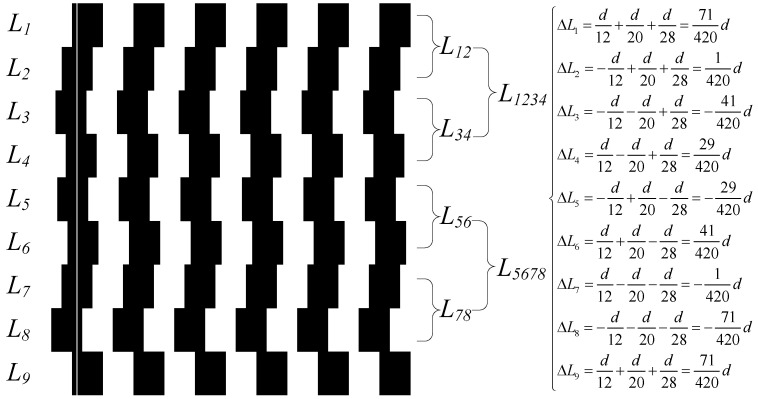
Phase-shift pattern of index grating and moving distance of each row.

In accordance with the above theory, the index grating’s phase-shift pattern is designed and illustrated in Figure 7. The black zones represent the chrome-plated area of the index grating, which is opaque, and the rest of the area is transparent. The white line in the first black column is the centerline before phase shift. In Figure 7, the grid lines are divided into nine rows: *L*_1_, *L*_2_…*L*_9_. The right-moving distances of the rows are Δ*L*_1_, Δ*L*_2_…Δ*L*_9_, and the height of each row is H/8, where H is the height of the incremental track detector. Row *L*_1_ can be regarded as moving x−d/20−d/28 to the right by d/12, and row *L*_2_ can be regarded as moving x−d/20−d/28 to the left by d/12. Therefore, the third-order harmonic can be suppressed with the superposition of signals generated by *L*_1_ and *L*_2_. Similarly, with *L*_3_ and *L*_4_, *L*_5_ and *L*_6_, and *L*_7_ and *L*_8_, the third-order harmonic can be suppressed. After the third-order harmonic is suppressed, the combination of *L*_1_ and *L*_2_, namely *L*_12_, can be regarded as moving x−d/28 to the right by d/20, and the combination of *L*_3_ and *L*_4_, namely *L*_34_, can be regarded as moving x−d/28 to the left by d/20. Therefore, the fifth-order harmonic can be suppressed with the superposition of signals generated by *L*_12_ and *L*_34_. Analogically, with *L*_56_ and *L*_78_, the fifth-order harmonic can also be suppressed. After the third- and fifth-order harmonics are suppressed, the combination of *L*_1_, *L*_2_, *L*_3_, and *L*_4_, namely *L*_1234_, can be regarded as moving x to the right by d/28, and the combination of *L*_5678_, can be regarded as moving x to the left by d/28. Therefore, the seventh-order harmonic can be suppressed with the superposition of signals generated by *L*_1234_ and *L*_5678_. Using this design, the third-, fifth-, and seventh-order harmonics are suppressed.

In general, dividing the index grating’s grid lines into eight rows is sufficient to suppress harmonics. The ninth row is added in consideration of installation and adjustment. Δ*L*_9_ = Δ*L*_1_. This is because the index grating’s incremental windows, as well as the photoelectric device’s incremental track detector, cannot be completely aligned in most cases. Adding this row on the index grating can leave a margin for position adjustments. In case there is a certain deviation in the vertical direction, the effect of suppressing harmonics will not be affected.

## 4. Experiments and Results

### 4.1. Experimental Setup

**Figure 8:**
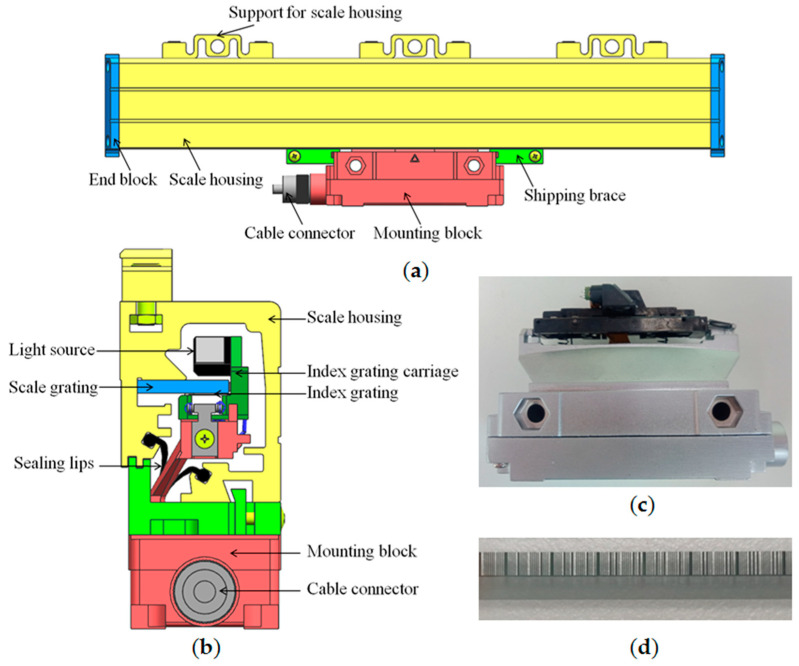
Structure of absolute linear encoder. (**a**) External structure; (**b**) internal structure; (**c**) scanning carriage; (**d**) part of scale grating.

Figure 8 shows the absolute linear encoder’s structure. The absolute linear encoder’s external structure is composed of a scale housing, end blocks, supports for scale housing, a mounting block, shipping braces, and a cable connector, as shown in Figure 8a. The absolute linear encoder’s internal structure is composed of an index grating carriage, a light source, an index grating, a scale grating, etc., as shown in Figure 8b. The absolute linear encoder can also be divided into two components. One is the scale housing component, including scale housing, end blocks, supports for scale housing, a scale grating, etc. The other is the scanning carriage component, including a mounting block, an index grating carriage, a light source, an index grating, a photoelectric device, internal circuit boards, etc. The real scanning carriage is illustrated in Figure 8c. Part of the scale grating is shown in Figure 8d. The absolute linear encoder actually measures the position through the relative movement of the scale housing and scanning carriage components.

**Figure 9:**
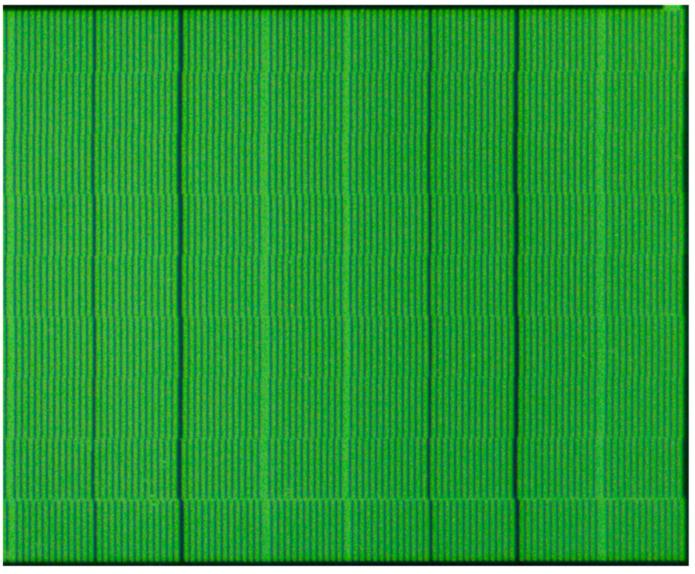
Part of incremental windows of fabricated index grating.

The index grating’s traditional incremental windows were engraved with grid lines of 20 μm grating. Figure 9 shows the part of the fabricated index grating’s incremental windows after applying the two methods proposed in Section 3. The arrangement of the index grating’s incremental windows has been modified, and each column has been shifted.

**Figure 10:**
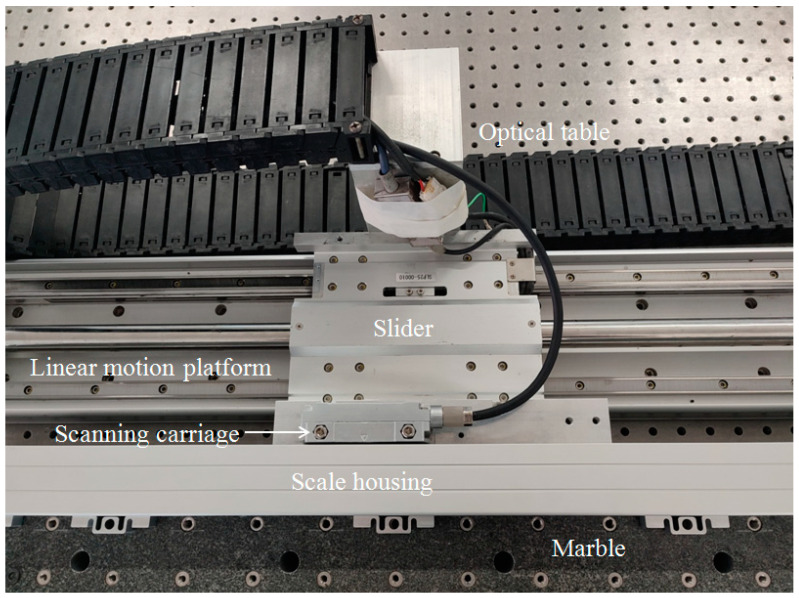
Experimental setup.

The position error over the linear encoder’s entire measuring range can be determined by laser interferometer [17,18]. The SDE of the linear encoder can be measured by the constant-speed approach [31], which we adopted in our experiments. The experimental setup, shown in Figure 10, was placed in a temperature- and humidity-controlled room. The scale housing of the tested absolute linear encoder was fixed on the marble, and the scanning carriage was fixed on the slider of the linear motion platform (SLP25-1000-S-M3-A3, NPM, Tokyo, Japan). The linear motion platform and the marble were fixed on the optical table.

### 4.2. Results and Discussion

**Figure 11:**
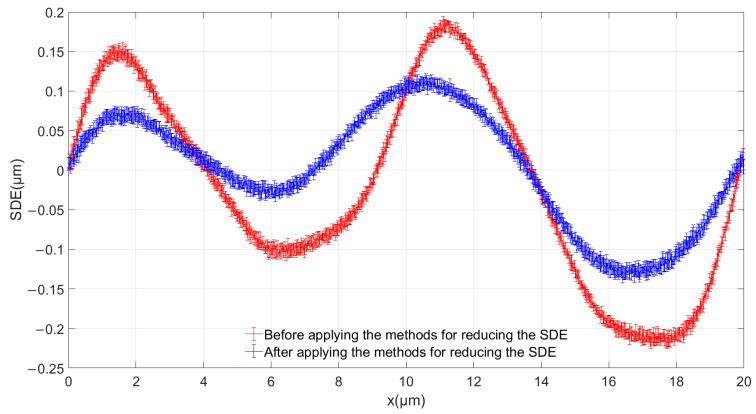
Comparison of SDE.

We set the speed of the linear motion platform to 100 mm/s, while the grating period of the absolute linear encoder’s incremental track was 20 μm, and the sampling frequency was 5 MHz, so there were 1000 sampling points in each period. The initial position corresponded to 0 μm, the last sampling point corresponded to 20 μm, and the distance between adjacent sampling points was 0.02 μm. The position of each sampling point can be regarded as the theoretical position of $x_{ideal}$, and $x_{real}$ can be obtained through Equation (4). Finally, the SDE can be obtained through Equation (5). In order to verify the reliability of the results, we obtained five periods before and after applying the methods for reducing the SDE. The five periods were different grating periods selected in a measurement range. Figure 11 shows the average results of the SDE curves, with the error bar representing the standard deviation. Before applying the methods for reducing the SDE, the SDE is within ±0.218 μm. After applying the methods for reducing the SDE, the SDE is within ±0.135 μm. The SDE has been decreased by 38.07%. We concluded that our proposed methods for reducing the absolute linear encoder’s SDE are effective.

The proportion of each higher-order harmonic ($A_{n}/A_{1}$) can be calculated using a fast Fourier transformation (FFT) analysis of the collected Moiré fringe signals, as shown in Table 1. Among them, we pay more attention to the proportion of the third-, fifth-, and seventh-order harmonics. After applying the methods for reducing the SDE, the proportion of the third-, fifth-, and seventh-order harmonics significantly decreases. In particular, the proportion of the third-order harmonic reaches 2.78% before applying our proposed methods, and such harmonic distortion is large and has a great influence on the sine of Moiré fringe signals. In contrast, the proportion is reduced to 0.06% with the new index grating, and the influence on the sine of Moiré fringe signals is alleviated.

## 5. Conclusions

In our paper, we studied an absolute linear encoder’s working principle. We proposed two methods for reducing the absolute linear encoder’s SDE, namely the single-field scanning method based on the shutter-shaped Moiré fringe and the method for suppressing harmonics through a phase shift of the index grating. Both methods are easily implemented without adding additional components. In our experiments, we measured the absolute linear encoder’s SDE with an approach based on constant speed, and we observed that the SDE decreased from ±0.218 μm to ±0.135 μm, which is a decrease of 38.07%. Our FFT analysis of the collected Moiré fringe signals demonstrated that the third-, fifth-, and seventh-order harmonics were effectively suppressed, confirming the feasibility of our proposed methods. In addition, we applied methods to reduce the SDE only from the perspective of reducing the impact of linear errors and harmonic distortion. For future research, advanced subdivision algorithms can be used to further reduce the impact of DC offset, amplitude, and phase-shift errors, which will further reduce the absolute linear encoder’s SDE and improve its performance.

## Figures and Tables

**Table 1:** Proportion of higher-order harmonics.

	$A_{2}/A_{1}$	$A_{3}/A_{1}$	$A_{4}/A_{1}$	$A_{5}/A_{1}$	$A_{6}/A_{1}$	$A_{7}/A_{1}$
Before applying the methods for reducing the SDE	0.30%	2.78%	0.17%	0.85%	0.11%	0.38%
After applying the methods for reducing the SDE	0.29%	0.06%	0.18%	0.04%	0.11%	0.02%

## Data Availability

Not applicable.

## Abbreviations

The following abbreviations are used in this manuscript:

SDE	Subdivision error within one signal period
FFT	Fast Fourier transformation
